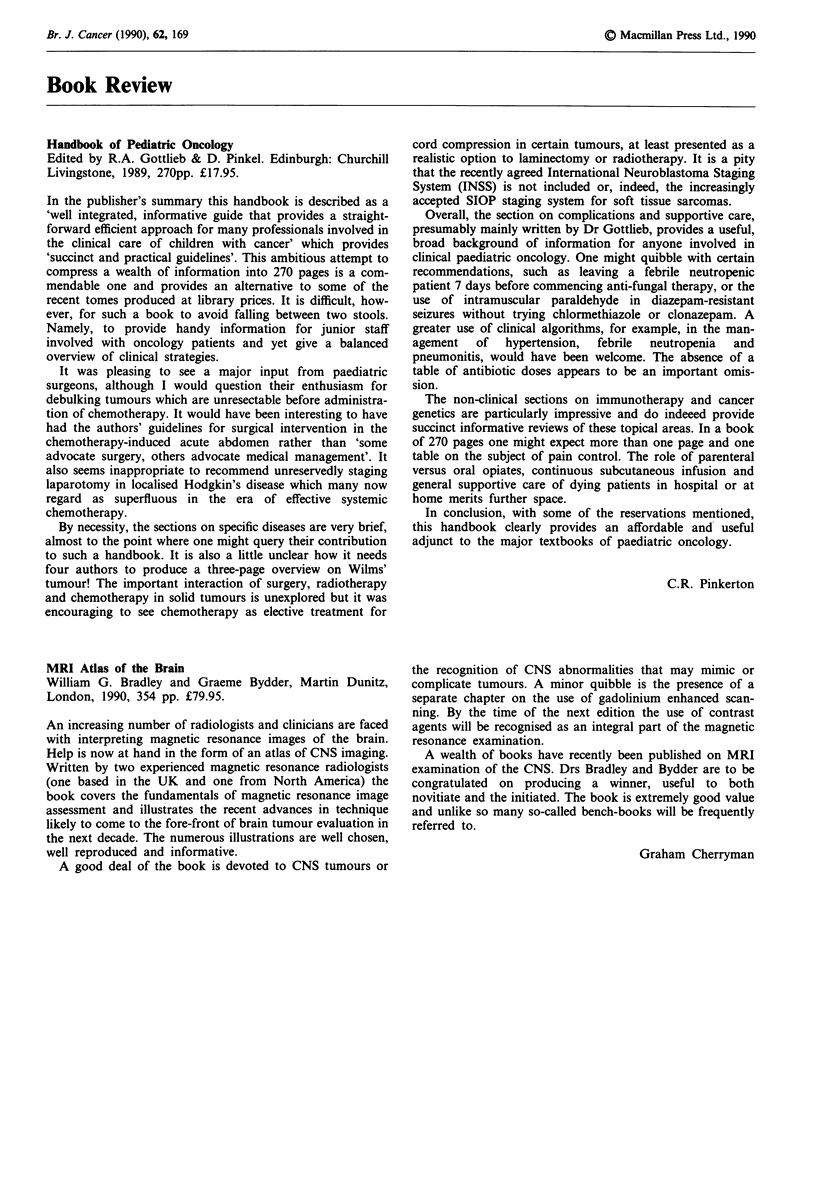# MRI Atlas of the Brain

**Published:** 1990-07

**Authors:** Graham Cherryman


**MRI Atlas of the Brain**
William G. Bradley and Graeme Bydder, Martin Dunitz, London, 1990, 354 pp. £79.95.

An increasing number of radiologists and clinicians are faced with interpreting magnetic resonance images of the brain. Help is now at hand in the form of an atlas of CNS imaging. Written by two experienced magnetic resonance radiologists (one based in the UK and one from North America) the book covers the fundamentals of magnetic resonance image assessment and illustrates the recent advances in technique likely to come to the fore-front of brain tumour evaluation in the next decade. The numerous illustrations are well chosen, well reproduced and informative.

A good deal of the book is devoted to CNS tumours or the recognition of CNS abnormalities that may mimic or complicate tumours. A minor quibble is the presence of a separate chapter on the use of gadolinium enhanced scanning. By the time of the next edition the use of contrast agents will be recognised as an integral part of the magnetic resonance examination.

A wealth of books have recently been published on MRI examination of the CNS. Drs Bradley and Bydder are to be congratulated on producing a winner, useful to both novitiate and the initiated. The book is extremely good value and unlike so many so-called bench-books will be frequently referred to.

Graham Cherryman